# Exome sequencing of 500 Brazilian patients with rare diseases: what we have learned

**DOI:** 10.1590/1516-3180.2022.0076.R1.21072022

**Published:** 2022-09-12

**Authors:** Caio Robledo D’Angioli Costa Quaio, Caroline Monaco Moreira, Christine Hsiaoyun Chung, Sandro Felix Perazzio, Aurelio Pimenta Dutra, Chong Ae Kim

**Affiliations:** I MD. Researcher and PhD Candidate, Instituto da Criança (ICr), Hospital das Clinicas HCFMUSP, Faculdade de Medicina FMUSP, Universidade de São Paulo, São Paulo, SP, BR; Medical Geneticist, Fleury Medicina e Saúde, São Paulo (SP), Brazil; Medical Geneticist, Laboratório Clínico, Hospital Israelita Albert Einstein (HIAE), São Paulo (SP), Brazil.; II MSc. Molecular Biology Leader, Fleury Medicina e Saúde, São Paulo (SP), Brazil.; III MD. Medical Geneticist, Fleury Medicina e Saúde, São Paulo (SP), Brazil.; IV MD, PhD. Immunology Division, Fleury Medicina e Saúde, São Paulo (SP), Brazil; Division of Rheumatology, Universidade Federal de Sao Paulo, Sao Paulo, Brazil; Central Laboratory, Hospital das Clinicas HCFMUSP, Faculdade de Medicina FMUSP, Universidade de São Paulo, São Paulo, SP, Brazil.; V MD, PhD. Neurology Division, Fleury Medicina e Saúde, São Paulo (SP), Brazil.; VI MD, PhD. Head of Genetics Unit and Associate Professor, Instituto da Criança (ICr), Hospital das Clinicas HCFMUSP, Faculdade de Medicina FMUSP, Universidade de São Paulo, São Paulo, SP, BR.

Dear Editor

Rare diseases comprise a large and diverse group of an estimated 7,000 different conditions that collectively affect millions of people worldwide. We recently studied the genomic findings of 500 Brazilian patients with suspected rare diseases of genetic etiology who have undergone exome sequencing (ES) for diagnostic purposes.^1^

**Figure 1.**
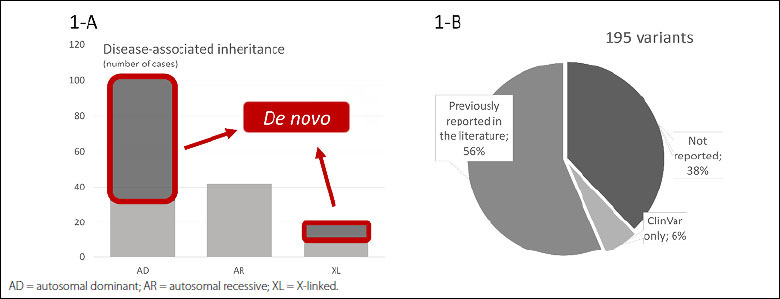
Inheritance pattern and variant characteristics. **A** shows the distribution of inheritance patterns of the 164 diagnoses and highlights the proportion of *de novo* events; exome sequencing was able to unravel the diagnosis in 158 patients (six patients presented dual molecular diagnoses), which represents an overall diagnostic yield of 31.6%. The 164 diagnoses comprised 101 autosomal dominant, 42 autosomal recessive, and 21 X-linked conditions. **B** shows the distribution of variants according to the literature (HGMD Professional Database) and ClinVar database.

We observed an overall diagnostic yield of 31.6% in our cohort. Figure 1–A shows the inheritance patterns of the genetic diseases. These diagnoses were associated with 195 sequence variants, among which 38% were rare variants that have not been previously published in the literature (Figure 1–B). The diagnostic rate varied widely depending on age, and we observed higher diagnostic rates in prenatal samples (67%) and children younger than one year (44%) and lower rates for adults older than 50 years (13%). Undiagnosed patients still comprise the majority of patients in our cohort (68.4%) and remain a challenge in genomics. Even with the advances in genomic technology, for many patients with rare diseases, the diagnostic odyssey has not come to an end. Valuable techniques such as trio exome analysis (testing of samples from a proband and both parents) or genome sequencing may increase the genetic diagnosis of rare diseases.

AD = autosomal dominant; AR = autosomal recessive; XL = X-linked.

**Figure 2.**
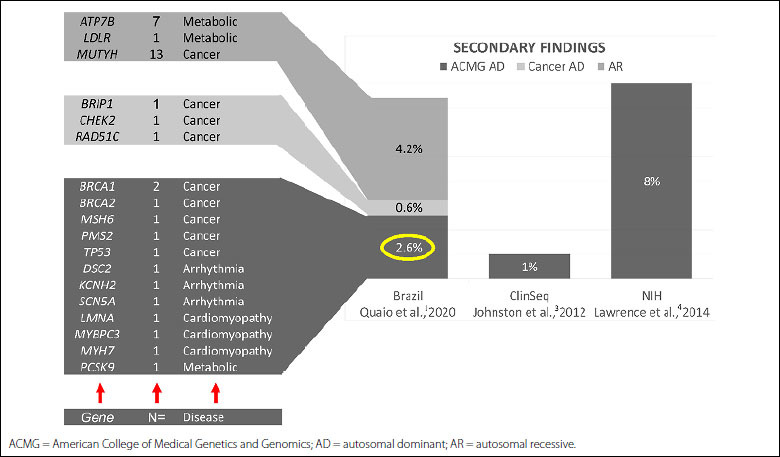
Secondary findings observed in our cohort of Brazilian patients compared with other studies in the literature. On the left, we observe the distribution of secondary findings: 37 patients (7.4%) presented a reportable secondary finding, among which 2.6% are those who harbored pathogenic or likely pathogenic variants associated with autosomal dominant diseases of obligatory report according to the American College of Medical Genetics and Genomics recommendations, 0.6% presented other autosomal dominant forms of hereditary cancer and 4.2% were carriers for variants associated with autosomal recessive diseases in American College of Medical Genetics and Genomics list of reportable secondary findings.

We also found additional genetic alterations that may directly affect the morbidity and mortality of individuals. In 37 patients (7.4%), we found deleterious genetic variants associated with clinically actionable conditions, such as hereditary cancer, arrhythmia, metabolic diseases, and cardiomyopathies. These secondary findings were previously referred to as “incidental findings”. (Figure 2).

ACMG = American College of Medical Genetics and Genomics; AD = autosomal dominant; AR = autosomal recessive.

Determining reportable secondary findings remains controversial and challenging.^2,3,4^ Discussions on this subject are prevalent in North American, European, and some Asian countries but have yet to take place in Brazil and other Latin American countries. Indeed, there are no regulatory documents, legislation, or policies from scientific societies in Brazil regarding the protocols for reporting secondary findings in genomic studies. We urge our medical societies to adopt specific policies for reporting these conditions, and more importantly, consider the Brazilian frequencies of rare diseases. We believe that our study has made an important contribution to the knowledge of rare diseases of genetic etiology in Brazil, a country very underrepresented on this subject.

We also studied the carrier status for recessive diseases in 320 symptomatic patients in this cohort.^5,6^ We found at least one pathogenic or likely pathogenic heterozygous variant associated with recessive diseases in the majority of individuals (71.9%). We believe that population studies of recessive diseases are important because recessive diseases are relatively frequent in aggregate, have a high clinical impact, early management can impact clinical outcomes, and some of them can be detected by neonatal screening (e.g., phenylketonuria).

In one of the largest cohorts of rare diseases in Latin America, we observed that ES was a powerful method for identifying the molecular basis of monogenic disorders, redirecting clinical care, and guiding health policies for rare diseases. We hope that our study will encourage others to better understand the clinical and social burden of rare genetic diseases in developing countries.
